# Enhancement of Biopolymer Film Properties Using Spermidine, Zinc Oxide, and Graphene Oxide Nanoparticles: A Study of Physical, Thermal, and Mechanical Characteristics

**DOI:** 10.3390/ma18020225

**Published:** 2025-01-07

**Authors:** Esmaeil Vafaei, Maryam Hasani, Nasrin Salehi, Farzaneh Sabbagh, Shirin Hasani

**Affiliations:** 1 Department of Food Science and Technology, Shahrood Branch, Islamic Azad University, Shahrood 3619943189, Iran; esmaeilvafaei1361@gmail.com; 2 Department of Basic Sciences, Shahrood Branch, Islamic Azad University, Shahrood 3619943189, Iran; salehi9002@gmail.com; 3 Department of Biosystems and Soft Matter, Institute of Fundamental Technological Research, Polish Academy of Sciences, 02-106 Warsaw, Poland; 4 Department of Fisheries, Faculty of Fisheries and the Environment, Gorgan University of Agricultural Sciences and Natural Resources, Gorgan 4913815739, Iran; shirin.hasani88@gmail.com

**Keywords:** nanocomposite, gelatin, chitosan, zinc oxide, graphene oxide

## Abstract

One of the main limitations of biopolymers compared to petroleum-based polymers is their weak mechanical and physical properties. Recent improvements focused on surmounting these constraints by integrating nanoparticles into biopolymer films to improve their efficacy. This study aimed to improve the properties of gelatin–chitosan-based biopolymer layers using zinc oxide (ZnO) and graphene oxide (GO) nanoparticles combined with spermidine to enhance their mechanical, physical, and thermal properties. The results show that adding ZnO and GO nanoparticles increased the tensile strength of the layers from 9.203 MPa to 17.787 MPa in films containing graphene oxide and zinc oxide, although the elongation at break decreased. The incorporation of nanoparticles reduced the water vapor permeability from 0.164 to 0.149 (g.m^−2^.24 h^−1^). Moreover, the transparency of the layers ranged from 72.67% to 86.17%, decreasing with higher nanoparticle concentrations. The use of nanoparticles enhanced the light-blocking characteristics of the films, making them appropriate for the preservation of light-sensitive food items. The thermal properties improved with an increase in the melting temperature (Tm) up to 115.5 °C and enhanced the thermal stability in the nanoparticle-containing samples. FTIR analysis confirmed the successful integration of all components within the films. In general, the combination of gelatin and chitosan, along with ZnO, GO, and spermidine, significantly enhanced the properties of the layers, making them stronger and more suitable for biodegradable packaging applications.

## 1. Introduction

The significant increase in plastic waste, coupled with the challenges of recycling these materials, has driven the food and packaging industries to explore biodegradable alternatives. Packaging plays a vital role in preserving the quality of food and raw materials by protecting them from oxidation, microbial spoilage, and extending the shelf life [1,2]. However, the widespread use of synthetic polymers, which are not fully biodegradable, has led to serious environmental concerns. Edible films and coatings have recently gained considerable attention due to their renewable nature and environmental compatibility. These biodegradable films are primarily composed of polysaccharides (such as chitosan, cellulose, alginate, and starch), proteins, and fats. Among polysaccharides, chitosan has been extensively studied in the food and pharmaceutical industries due to its film-forming capability and its unique mechanical, physical, and antimicrobial properties [3]. Chitosan is a linear polysaccharide derived from the deacetylation of chitin and is the second most abundant polysaccharide in nature after cellulose. The antimicrobial properties of chitosan are attributed to the presence of positively charged amino groups that interact with negatively charged cell membranes of microorganisms, leading to the leakage of proteins and intracellular components [4]. Gelatin, another key protein used in edible films, is derived from the breakdown of collagen. Gelatin has the unique ability to form reversible films at temperatures close to body temperature, making it particularly valuable in the food and medical industries. However, gelatin films tend to be brittle due to the formation of microcrystalline structures. To address this issue, plasticizers are often added to improve flexibility, impact resistance, and prevent cracking during packaging and transportation. Polysaccharide-based films generally exhibit better mechanical strength than protein-based films; however, their hydrophilic nature makes them permeable to water vapor [3]. To overcome this limitation, nanotechnology can be utilized to enhance the barrier properties of these films. Research has shown that bilayer films made from a combination of chitosan and gelatin exhibit improved mechanical and physical properties compared to films made from each material alone. This improvement is attributed to the formation of polyelectrolyte complexes resulting from electrostatic interactions between the ammonium groups of chitosan and the negatively charged side groups of gelatin or collagen chains. In addition to improving the mechanical properties, the antimicrobial activity of chitosan films can be further enhanced by incorporating nanoparticles such as titanium dioxide, magnesium oxide, copper oxide, zinc oxide, and silver [5,6]. Among these, zinc oxide is one of the most widely used mineral oxides due to its chemical stability, environmental compatibility, UV resistance, and low cost. Zinc oxide also has proven antimicrobial properties, particularly in controlling airborne diseases. Zinc oxide (ZnO) is recognized as safe by regulatory authorities such as the U.S. Food and Drug Administration (FDA). Additionally, studies conducted by the European Food Safety Authority (EFSA) have indicated that ZnO, when present in nanoform, does not migrate into food products. Based on its evaluation, the EFSA recommends a permissible limit for ZnO in food ranging from 5 to 25 mg/kg of food [7,8]. Graphene and graphene oxide have also attracted significant attention in recent years for their unique properties, including antimicrobial activity. Graphene oxide, with its carboxyl and hydroxyl groups, interacts with bacterial cell membranes, leading to their destruction [9]. To further enhance the physicochemical properties of edible films, plasticizers such as spermidine have been shown to improve the mechanical and barrier properties of protein-based films [10,11,12]. Spermidine, particularly when combined with glycerol, can significantly enhance the functional properties of chitosan–gelatin films, making them viable alternatives to synthetic polymers commonly used in the food packaging industry [13,14]. This study aims to prepare and evaluate chitosan–gelatin films incorporated with zinc oxide and graphene oxide nanoparticles, as well as spermidine, to improve their physical, mechanical, and antimicrobial properties. The goal is to develop biodegradable packaging materials that can mitigate the environmental impact of non-biodegradable plastics.

## 2. Materials and Methods

In this research, gelatin powder (Sigma-Aldrich, St. Louis, MO, USA), zinc oxide (ZNO) nanoparticles (Sigma-Aldrich, St. Louis, MO, USA), graphene oxide (GO) graphene oxide (GO), and commercial chitosan (with medium molecular weight) were used. Glycerol (about 87%) was supplied from the Merck Chemical Company (Darmstadt, Germany), whereas spermidine was from Sigma-Aldrich Company (St. Louis, MO, USA). All other chemicals were analytical grade.

### 2.1. Preparation of Gelatin–Chitosan Film

To prepare the gelatin–chitosan film, gelatin powder was completely dissolved in distilled water at 35 °C. Chitosan biopolymer solution was specifically prepared using commercial chitosan powder (2 g) in 1% acetic acid (100 mL). Then, the chitosan solution was slowly added to the above gelatin solution to form the chitosan–gelatin polyelectrolyte and the solution was stirred for 6 h using a magnetic stirrer at a speed of 600 rpm. Then, glycerol with a concentration of 30% *w*/*w* and spermidine with a concentration of 30% *w*/*w* were added to the solution as softeners and plasticizers to increase flexibility and reduce brittleness [13]. The solution of ZNO nanoparticles with a concentration of 5% *w*/*w* and GO with a concentration of 3% *w*/*w* was dissolved and heated at 62 °C with continuous stirring for 1 h and then without heat on the shaker. It was mixed to produce a homogeneous solution. To ensure homogeneity of nano solutions, the solutions were homogenized in an ultrasonic bath for one hour [15]. Then, the prepared ZNO and GO nanoparticles were added drop by drop to the prepared chitosan–gelatin solution and the resulting solution was molded in a special container and placed in a vacuum oven for 12 h to dry. The film containing metal nanoparticles, spermidine, and dried glycerol was separated from the mold and subjected to different analyses. The present study included 5 treatments: control film (chitosan–gelatin), chitosan–gelatin film containing spermidine, chitosan–gelatin film containing spermidine and nano zinc oxide, chitosan–gelatin film containing spermidine and GO, chitosan–gelatin film containing spermidine, zinc nano oxide, and graphene nano oxide.

### 2.2. Physical and Mechanical Properties of Films

Films were packed in zippered bags and placed in a saturated magnesium nitrate-containing desiccator (RH 50% and at 25 °C) during analysis.

#### 2.2.1. Film Thickness

A digital micrometer (with an accuracy of 0.001 mm, Mitutoyo Corporation, Berlin, Germany) was used to measure the thickness of the films. Measurements were repeated randomly at five points in each film. The average thickness of these points was used to determine the physical and mechanical properties of the films [16].

#### 2.2.2. Mechanical Properties

Tensile strength (TS) and elongation at break (EAB) were evaluated using a texture analyzer (CT3, Brookfield Engineering Laboratories, Inc., Middleboro, MA, USA). Film strips with dimensions of 20 × 50 mm were securely placed in sample grips, with an initial grip separation of 30 mm and a stretching rate of 30 mm/min. The applied force and displacement were continuously recorded as the film was elongated until rupture. Each film sample was subjected to a minimum of five independent tests [17]. The TS and EAB were determined using the following equations:

$$\mathrm{TS}\;(\mathrm{MPa})=\frac{F}{LM}$$

where F (N) is the maximum tension when the film broke, L (mm) is the thickness of the film, and M (mm) is the width of the film.

$$\mathrm{EAB}\;(\%)=\frac{L1-L0}{L0}\times 100$$

where L1 is the length of the elongation during the film fracture (mm) and L0 is the initial length of the film (mm).

#### 2.2.3. Water Solubility

The water solubility of gelatin–fucoidan films was measured according to Roshandel-hesari et al. [18]. The films (2.5 × 2.5 cm) were first dried in an oven at 105 °C for 24 h to obtain a constant initial weight. The dried samples were then placed in containers containing 50 mL of distilled water for 24 h. They were filtered through Whatman filter paper no. 1. The residue was separated from the filter paper and weighed after drying in an oven at 105 °C for 24 h. The solubility of the samples was calculated according to the following formula:

$$\mathrm{Solubility}\;(\%)=\frac{initial\;film\;weight-final\;dried\;film\;weight}{initial\;film\;weight}\times 100$$

#### 2.2.4. Water Vapor Permeability (WVP)

The water vapor permeability (WVP) of the films was determined by securely placing them over the top of permeation cells containing 10 mL of distilled water at 100% relative humidity (RH), resulting in a vapor pressure difference of 2.337 × 10^3^ Pa at 20 °C. The amount of water vapor passing through the films was quantified by measuring the weight loss of the permeation cells over an 8 h period, following the method outlined by Poverenov et al. [19]. Each measurement was performed in triplicate. The WVP was then calculated using the following equation:

WVP = W.X/A.T.Δ

where W represents the weight loss (g), X is the film thickness (m), A is the exposed film area (m^2^), T is the time (s), and Δp is the vapor pressure difference (Pa).

#### 2.2.5. Color and Evaluation of Optical Properties of Films

A colorimeter (BYK Gardner Model) was used to determine the color of the prepared films. The films were placed on a standard white screen (L* = 94.63, a* = −0.88, b* = 0.65). The color parameters such as L* (lightness/brightness), a* (redness/greenness), and b* (yellowness/blueness) values were recorded by 5 replicates. ∆E (total color difference values of the film were calculated) as reported by Lanier et al. [20].

### 2.3. Scanning Electron Microscopy (SEM)

Morphology of the films was displayed using a scanning electron microscope (Philips XL30, Eindhoven, The Netherlands). Films were cut into 3 × 3 cm and glued to an aluminum base with silver glue. The bases were coated with gold in a coating/spraying apparatus. The surface of the samples was imaged at different magnifications [21].

### 2.4. Fourier Transform Infrared Spectroscopy

Fourier transform infrared spectroscopy (FTIR) spectra of films were determined using FTIR Spectrometer (Perkin-Elmer, Spectrum Rxi, Waltham, MA, USA) in the range of 400 to 4000 cm^−1^ and in the resolution of 4 cm^−1^ [22].

### 2.5. X-Ray Diffraction Analysis (XRD)

The XRD patterns were recorded using a Bruker D8 Advance diffractometer (Billerica, MA, USA) equipped with a 3 kW generator, using nickel-filtered Cu Ka radiation (Malvern, UK) (k = 0.15418 nm) operating at 40 kV and 30 mA and a LYNXEYE detector [22].

### 2.6. Differential Scanning Calorimetry (DSC)

The thermal properties of the samples were measured using a differential scanning calorimeter (Shimadzu model, Kyoto, Japan). An amount of 0.3 g of the sample was scanned at a speed of 10 °C/min in the temperature range of 0° to 300°. From the obtained thermogram, melting temperatures (Tm) and glass transition temperature (Tg) were determined [23].

### 2.7. Statistical Analysis

Statistical analyses were conducted using SPSS software (Version 26). A one-way analysis of variance (ANOVA) was employed, followed by Duncan’s multiple range test for mean comparisons. Statistical significance was set at *p* < 0.05, with significant differences indicated by distinct letters.

## 3. Results and Discussion

### 3.1. Thickness of Gelatin–Chitosan Films

All the produced films showed good flexibility and smooth surfaces. As shown in Table 1, the thickness of the films varied between 0.14 and 0.171, and with the increase in nanoparticles, the thickness of the films significantly increased. Among the prepared films, the control film had the lowest thickness, while the gelatin–chitosan film containing ZnO nanoparticles and GO had the highest thickness. This rise in thickness may be linked to the positioning of nanoparticles inside the network’s spaces, which results in a heterogeneous structure and increases the overall thickness. Other research observed similar findings, demonstrating that adding oxide nanoparticles increases the thickness of gelatin–chitosan-based films [24]. Our results showed spermidine can contribute to an increase in the thickness of gelatin–chitosan films in terms of its ability to create additional cross-linking among polymer chains. Kim et al. [25] found that these cross-links lead to a more compact and dense structure, which can result in an increase in the film thickness. Spermidine contributed to the increase in thickness by promoting cross-linking among the polymer chains, which resulted in a more compact structure.

### 3.2. Evaluation of Mechanical Properties

Tensile strength is the maximum stress a material can withstand without permanent deformation. Table 1 shows that adding ZnO, GO, and spermidine significantly increased the tensile strength of gelatin–chitosan films. The film’s structure is reinforced by the uniform dispersion of nanoparticles throughout the matrix, which facilitates the formation of ionic interactions between the nanoparticles and polymer chains. This finding aligns with previous research, indicating that ZnO and GO nanoparticles enhance the tensile strength of films by reinforcing the polymer matrix [26,27]. Spermidine further improves the inter-action between the polymer and nanoparticles, leading to a stronger composite structure.

**Figure 1:**
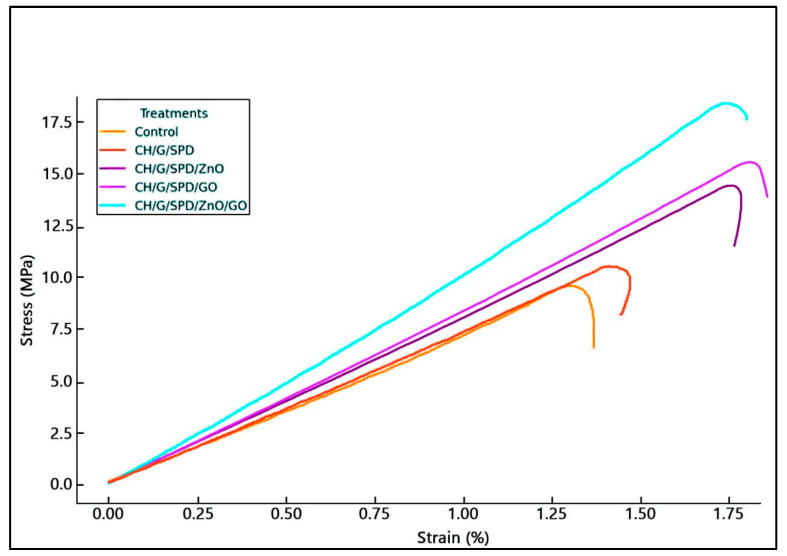
Stress–strain curves of CH/G films.

Elongation at break, which reflects flexibility, was affected by nanoparticle addition. Films containing ZnO and GO exhibited a lower elongation at break in terms of their reinforcing effect, which made the films stiffer and less flexible [28,29]. Spermidine had a less noticeable impact on elongation, but via altering the polymer interactions, it probably helped to enhance flexibility [14]. The elastic modulus (Young’s modulus) of the films increased with the incorporation of nanoparticles, as shown in Table 1. The highest modulus was observed in films containing ZnO and GO, indicating greater stiffness (Figure 1). This enhancement is in terms of ionic and hydrogen bonding between nanoparticles and the polymer matrix, which improves the film’s rigidity [30]. Spermidine improved the modulus by promoting the uniform distribution of nanoparticles, preventing agglomeration, and enhancing the film stiffness. Furthermore, ZnO and GO nanoparticles enhanced the resistance effect of the films. ZnO strengthens the polymer matrix by forming strong bonds with the polymer chains, increasing the tensile strength and Young’s modulus. Similarly, GO enhances the tensile strength by facilitating efficient stress distribution and improving film rigidity [31,32].

### 3.3. Moisture Content

Moisture content is critical for maintaining the mechanical properties and stability of films. Insufficient moisture can result in brittleness and a lack of flexibility, while excess moisture can result in decomposition and a reduction in the mechanical properties [33]. The moisture content of the gelatin–chitosan films was substantially reduced by the addition of nanoparticles, as demonstrated in Table 2. This decrease is due to nanoparticles filling the voids among the biopolymers, thus reducing the film’s ability to retain moisture. Zinc oxide (ZnO) acts as a moisture absorbent, improving the stability of the film by preventing moisture-related degradation. Graphene oxide (GO) further reduces the moisture content in terms of its low moisture absorption properties [34]. Thus, spermidine enhances the structural integrity of films, leading to reduced moisture absorption by forming stronger bonds between gelatin and chitosan molecules.

### 3.4. Solubility

Solubility is an important property for biodegradable films, especially in packaging wet products. The solubility of films with added nanoparticles was significantly lower than that of the control film (*p* ≤ 0.05). Specifically, the solubility decreased from 35.21% in the control film to 21.7 ± 0.06% in films containing ZnO and GO nanoparticles. This reduction is attributed to the dispersion of nanoparticles throughout the matrix, which helps cover hydrophilic sites and decreases moisture absorption [35]. Film stability is improved, and solvent solubility is diminished by the presence of ZnO nanoparticles, which are characterized by their size and chemical interactions with the polymer matrix. In the same vein, the film’s resistance to dissolution is enhanced by the layered structure of GO and its robust interactions with the matrix [36]. Spermidine, however, may slightly increase solubility, which can be advantageous for specific applications like food packaging where higher solubility might be desired.

### 3.5. Water Vapor Permeability (WVP)

**Figure 2:**
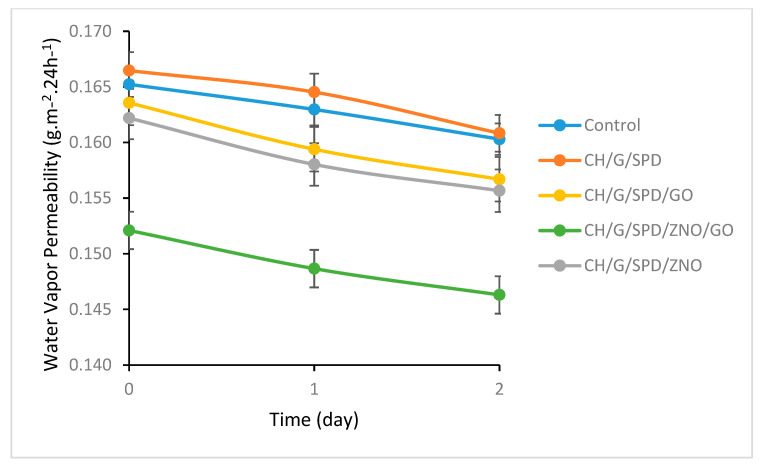
Water vapor permeability of CH/G films.

Water vapor permeability (WVP) plays a crucial role in determining the shelf life of food products. Effective moisture regulation is essential to maintain product quality during storage [37]. As shown in Figure 2, the incorporation of nanoparticles significantly reduced the WVP of the gelatin–chitosan films. This reduction is attributed to the uniform dispersion of nanoparticles with a high aspect ratio within the polymer matrix, which forms a physical barrier against moisture diffusion. ZnO nanoparticles, in particular, act as a physical barrier that hinders the penetration of external substances, making the films suitable for packaging applications where low permeability is critical. Furthermore, the nanoscale layered structure of GO functions as an effective barrier against gases and vapors, thereby contributing to reduced permeability. Strong interactions between GO and the polymer matrix, including hydrogen bonding and π-π interactions, further enhance the mechanical strength and impermeability of the films. The presence of spermidine improves the structural integrity of the films, reducing void spaces and preventing moisture and liquid infiltration. These findings are consistent with previous studies that demonstrate how GO’s high aspect ratio and surface area improve the barrier properties of gelatin–chitosan films by forming a labyrinth-like structure that gas and water vapor molecules must traverse, thus lowering permeability [38].

### 3.6. Swelling Studies

**Figure 3:**
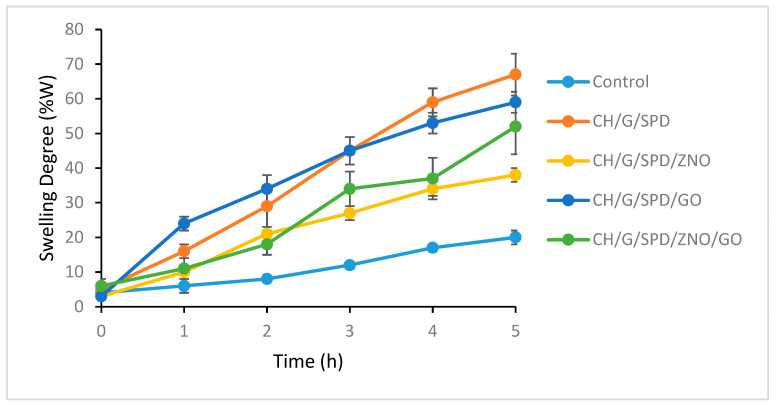
Swelling degree of CH/G films.

The swelling behavior of the films is shown in Figure 3. Adding spermidine to the gelatin–chitosan matrix promotes the formation of a more interconnected network, limiting swelling by reducing the water molecule space. Likewise, adding nanoparticles like ZnO and GO helps to lower edema. The barrier capabilities, cross-linking actions, and hydrophobic character of ZnO as well as the mechanical reinforcement given by GO [38] account for this result. Proper dispersion of GO within the matrix enhances uniformity and further reduces swelling. However, GO agglomeration may lead to inconsistent swelling behavior. When ZnO, GO, and spermidine are combined, their effects on swelling can be synergistic or competitive, depending on their interactions and concentrations. Increased cross-linking from these additives leads to a more compact polymer network, minimizing swelling. However, higher concentrations or poor dispersion of GO could increase water absorption, counteracting the swelling-reducing effects of ZnO and spermidine [33]. Previous studies confirm that ZnO nanoparticles, when uniformly dispersed in the matrix, function as physical barriers, increasing the path length for water molecules and reducing permeability [13].

### 3.7. The Values of L, a*, and b* of the Films

Color is an important attribute for packaging films, influencing their visual appeal and marketability. Nanofilms that are more transparent and resemble conventional syn-thetic plastics tend to be more accepted in the market, as they allow consumers to clearly see the packaged product [39]. Nevertheless, in order to prevent photooxidation and the degradation of nutritional quality, light-sensitive food products necessitate packaging materials that block light. Consequently, it is imperative to incorporate bio-enhancing compounds that also provide color in order to maintain the quality of food and attract consumers [40]. The color measurements for the films are shown in Table 3. The L values (lightness) ranged from 72.67 to 86.17, with the a* value indicating a shift towards redness (positive values) or greenness (negative values), and the b* parameter indicating a shift towards blue (negative values) or yellow (positive values). Gelatin naturally has a bluish hue, but when combined with chitosan, the color shifts towards yellow due to the inherent yellow tint of chitosan. The inclusion of ZnO nanoparticles amplified this yellow shift, and the ZnO nanoparticles introduced a slightly white or opaque appearance. GO, with its natural opacity, contributed to the darkening of the films, and the degree of darkening was influenced by the concentration and dispersion of GO within the matrix. The visual attractiveness of the films for packaging may be impacted by the increased opacity and darkening caused by high concentrations or inadequate dispersion of GO [41]. The results show that adding metal nanoparticles reduced the films’ transparency, likely in terms of an increased solid content in the films. The ZnO nanoparticles contributed to this opacity, but also improved the UV-blocking capabilities, offering better protection for the contents. The GO nanoparticles further reduced the transparency, and when combined with ZnO, the films achieved a balance between transparency and UV protection. While spermidine did not directly affect the optical properties, it improved the dispersion of nanoparticles like ZnO and GO, reducing particle aggregation and helping maintain or adjust the transparency levels in the composite films.

### 3.8. X-Ray Diffraction (XRD)

**Figure 4:**
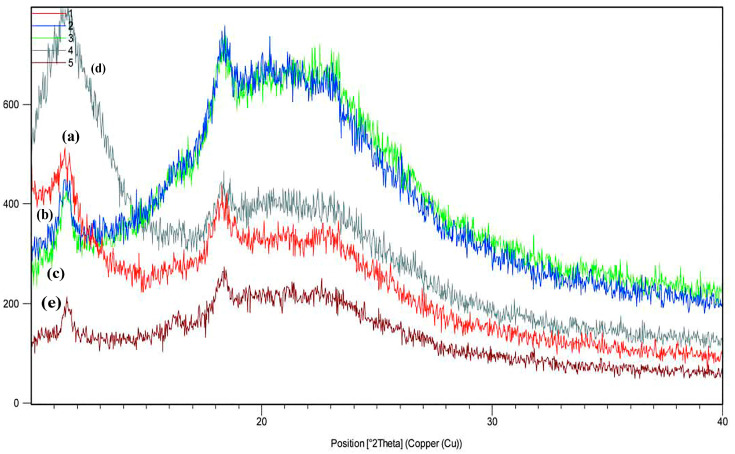
XRD patterns of (**a**) CH/G film (control), (**b**) CH/G/SPD film, (**c**) CH/G/SPD/ZnO film, (**d**) CH/G/SPD/GO, (**e**) CH/G/SPD/ZnO/GO film.

X-ray diffraction (XRD) analysis is a valuable tool to examine the crystalline structure of materials. In Treatment A, the peak intensities are lower than in other treatments, and several low-intensity peaks are observed at angles between 15 and 25 degrees, which could indicate lower crystallinity or an amorphous structure. In Treatment B, a significant peak emerges in the 18 to 20-degree range, indicating that the chitosan–gelatin film with spermidine has a more organized crystalline structure than the chitosan–gelatin alone. The samples containing ZnO show a larger, more distinct peak, indicating better crystallinity or a more pronounced crystalline phase. The highest peak intensity was observed in the treatment containing GO, showing a highly regular crystalline phase and the best crystalline structure. In the figures, two diffraction peaks can be seen at 11.5° and 18.5°, with 11.5° related to the diffraction of the 110 planes of the hydrated chitosan crystal structure, but generally, the peak near 18.5 is related to the amorphous state of chitosan. The absence of nanoparticle peaks may be attributed to the uniform and effective distribution and exfoliation of the layers. A unique peak is typically observed at approximately 10–12° for GO, which is indicative of its layered structure and corresponds to the (001) plane. The incorporation of ZnO and GO into the gelatin–chitosan film matrix can significantly affect the overall crystallinity of composite films. Sharp and intense peaks in the XRD pattern suggest a high degree of crystallinity, while broad and diffuse peaks are indicative of a more amorphous structure. By comparing the XRD patterns of the composite films with those of the pure gelatin and chitosan films, the effect of the nanoparticles on the crystallinity of the film can be evaluated. Shifts in the peak positions or variations in the peak intensity can reveal interactions between ZnO/GO and the gelatin–chitosan matrix. A slight alteration in the diffraction peaks of ZnO or variations in their strength may signify that the ZnO nanoparticles are uniformly disseminated throughout the polymer matrix or imply molecular interactions between the nanoparticles and the film constituents. These shifts or changes reflect how ZnO and GO affect the structural organization of the polymer matrix and its crystallinity [42]. The formation of new peaks or the disappearance of present ones may signal the creation of new crystalline phases or strong interactions between ZnO, GO, and the gelatin–chitosan matrix. These changes suggest alterations in the structural arrangement and crystallinity of the composite film in terms of the incorporation of GO [38]. The synergistic interactions between ZnO and GO can contribute to enhanced material properties, such as mechanical strength or barrier function, by affecting the overall crystallinity of the film. Furthermore, spermidine may affect the crystallinity of gelatin–chitosan films by altering the XRD pattern. Spermidine may interact with ZnO and GO nanoparticles, affecting their distribution and dispersion within the polymer matrix [27,36] (Figure 4).

### 3.9. FTIR Spectroscopy

**Figure 5:**
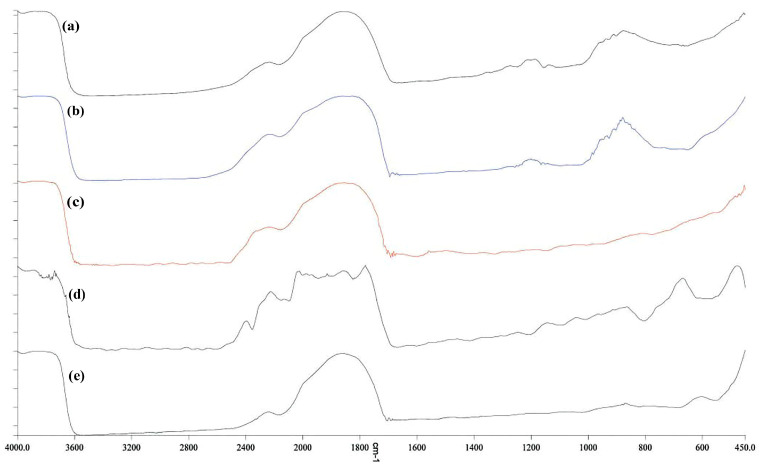
FTIR spectroscopy for (**a**) CH/G film (control), (**b**) CH/G/SPD film, (**c**) CH/G/SPD/ZnO film, (**d**) CH/G/SPD/GO, (**e**) CH/G/SPD/ZnO/GO film.

In Figure 5, the chemical structure of the films based on gelatin–chitosan and gelatin–chitosan containing nanoparticles was analyzed by comparing the peaks of each treatment. FTIR (Fourier transform infrared spectroscopy) spectra were used to examine the functional groups and interactions within the materials. Characteristic peaks of gelatin include amide I (around 1650 cm^−1^, due to C=O stretching), amide II (around 1550 cm^−1^, due to N-H bending and C-N stretching), and amide III (around 1240 cm^−1^, due to C-N stretching and N-H bending). For chitosan, the broad N-H stretching vibration appears around 3200–3400 cm^−1^, C-O stretching is seen around 1150–1060 cm^−1^, and C=O stretching in the amide group is observed around 1650 cm^−1^. Because of its interaction with the gelatin–chitosan matrix, the polyamine spermidine may contribute peaks linked to N-H stretching and C-H bending vibrations, which are represented as new or shifted peaks in the FTIR spectra. While ZnO nanoparticles do not show strong characteristic peaks, their interaction with the polymer matrix can be inferred from shifts or changes in the intensity of peaks associated with the polymer’s functional groups, such as N-H or C=O stretching vibrations. GO shows characteristic peaks like C=O stretching (around 1750 cm^−1^, due to carboxyl groups), O-H stretching (around 3700 cm^−1^), and C=C stretching (around 1650 cm^−1^ from aromatic rings). The incorporation of spermidine, ZnO, and GO into the gelatin–chitosan matrix may result in new peaks or shifts in the FTIR spectrum, indicating chemical interactions and hydrogen bonding between the additives and the polymer. These changes provide insights into the altered chemical structure and interactions within the film [38]. By analyzing FTIR spectra, researchers can understand the chemical composition and interactions in gelatin–chitosan films with spermidine, ZnO nanoparticles, and GO, enabling the tailoring of film properties for applications, such as packaging or biomedical uses [30].

### 3.10. Differential Scanning Calorimetry (DSC)

**Figure 6:**
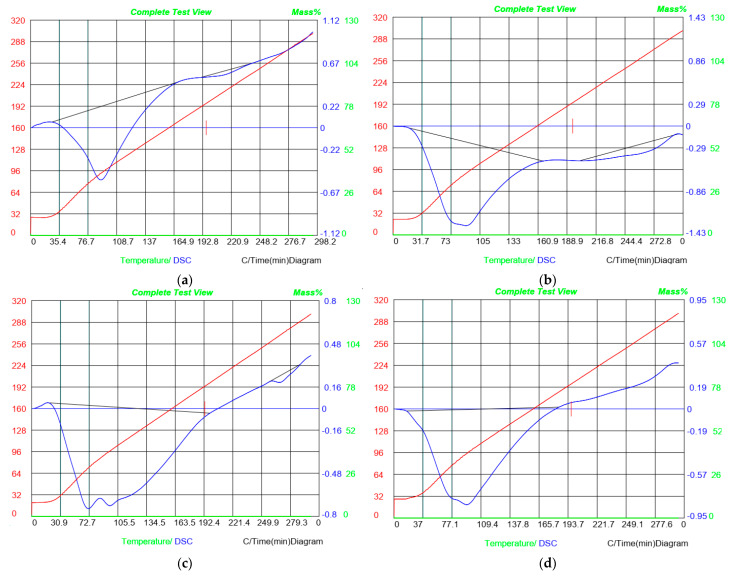

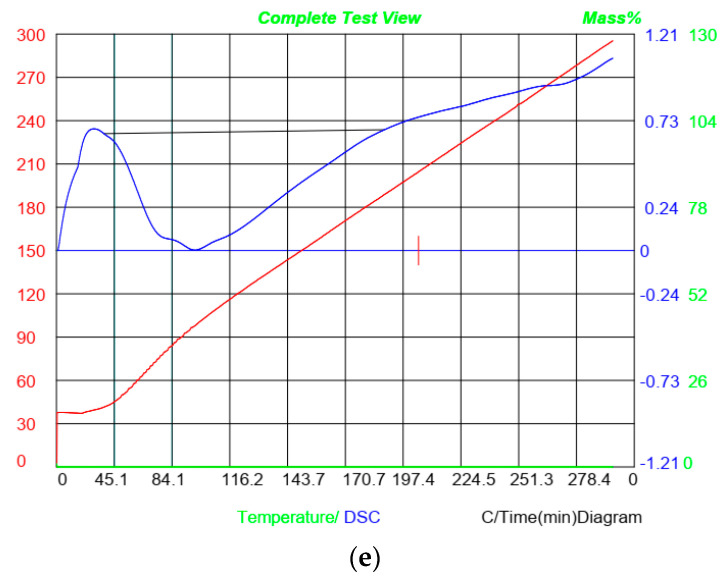
Differential scanning calorimetry (DSC) for (**a**) CH/G film (control), (**b**) CH/G/SPD film, (**c**) CH/G/SPD/ZnO film, (**d**) CH/G/SPD/GO, (**e**) CH/G/SPD/ZnO/GO film.

The thermal properties of the gelatin–chitosan-based films were evaluated in terms of the glass transition (Tg) and melting temperature (Tm) parameters, as shown in Figure 6. The glass transition temperature (Tg) represents the temperature at which the film transitions from a glassy to a rubbery state. DSC analysis revealed an endothermic peak in the gelatin–chitosan films corresponding to a melting temperature of 99.15 °C, with Tg observed at lower temperatures, ranging from 28.7 to 99.15 °C. In Treatment B, thermal changes begin at 23.4 °C, and the sample completely melts at 94.8 °C. Thus, a second thermal transition occurs between approximately 200 °C and 297 °C, indicating a chemical reaction or phase change. Tg varies from 21.6 to 100 °C for ZnO-containing films, whereas the endothermic melting peak is at 109 °C. Films comprising ZnO and GO undergo thermal changes beginning at 41.5 °C and melting at 115.5 °C. After 188.8 °C, no more thermal changes were seen. Similarly, in GO-containing films, the melting process ends at 185.5 °C, with no evidence of post-melting transitions such as crystallization. The presence of ZnO and GO nanoparticles significantly affects Tg, suggesting enhanced polymer network reinforcement and reduced polymer chain mobility in terms of strong interactions between nanoparticles and the polymer matrix [8,40,41]. Similarly, spermidine contributes to an increase in Tg, reflecting the reinforcement of the polymer network. Variations in Tm indicate the improved alignment and interaction of nanoparticles within the polymer matrix [32]. DSC data show enhanced thermal stability in films containing ZnO and GO nanoparticles, as seen in Figure 6. While those with ZnO and GO have the greatest melting temperatures, gelatin–chitosan films without nanoparticles show the lowest melting temperature. These results suggest the formation of stronger bonds within the polymer matrix, improving the mechanical and thermal properties of the films [21].

### 3.11. Surface Morphology

**Figure 7:**
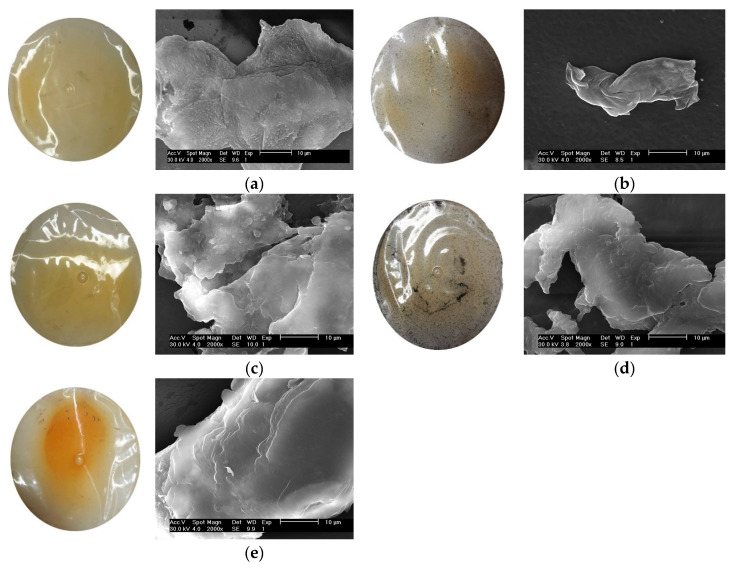
SEM images for (**a**) CH/G film (control), (**b**) CH/G/SPD film, (**c**) CH/G/SPD/ZNO film, (**d**) CH/G/SPD/GO, (**e**) CH/G/SPD/ZNO/GO film.

The effect of spermidine, ZnO, and GO on the surface morphology of the gelatin–chitosan films, as observed through scanning electron microscopy (SEM), is shown in Figure 7. The chitosan–gelatin control film’s SEM images demonstrate an uneven surface characterized by crystalline deposits and chitosan particles. The incorporation of ZnO into the chitosan–gelatin matrix produces a porous structure with a uniform distribution of ZnO particles. This transformation likely arises from interactions between ZnO nanoparticles and the chitosan polymeric chains, facilitating pore formation, even though pure chitosan films typically lack porosity. In films containing ZnO and graphene oxide (GO), the SEM images show some random pores and noticeable cracks. Moreover, despite the porous nature of these films, water vapor and gas permeability decrease. This can be explained by the non-interconnected nature of pores, which do not span the entire membrane. Consequently, the overall density of the membrane increases slightly, leading to reduced permeability. Thus, the SEM images of ZnO- and GO-containing films reveal smooth surfaces with no significant cracks or pores, though minor agglomerations of nanoparticles are visible. This structure contributes to the gradual reduction in permeability, as noted by Ludmila Motelica et al. [42]. Spermidine, a polyamine, improves hydrogen bonding and establishes more durable structures within the gelatin–chitosan matrix. As demonstrated by the SEM images [34,41], its incorporation substantially enhances the surface integrity and reduces the surface porosity, resulting in fewer defects and an improved mechanical strength. GO contributes to layered, homogeneous surface structures, attributed to its uniform distribution within the matrix and stronger bonds between gelatin and chitosan molecules. These structural changes improve the overall performance of the films. In general, the addition of spermidine, ZnO, and GO to gelatin–chitosan films enhances the mechanical properties, reduces surface defects, and forms more homogeneous and robust structures. These modifications expand the films’ potential applications in food packaging and biomedical fields.

## 4. Conclusions

This study demonstrated the successful enhancement of gelatin–chitosan films through the incorporation of ZnO nanoparticles, graphene oxide (GO), and spermidine. These additives significantly impacted various properties of the films, including their mechanical strength, moisture content, solubility, water vapor permeability (WVP), and swelling behavior. The inclusion of ZnO and GO nanoparticles notably improved the tensile strength and elasticity of the films, which can be attributed to the uniform dispersion and strong interactions between the nanoparticles and the polymer matrix. Spermidine further contributed to film reinforcement by facilitating cross-linking between the polymer chains, thus enhancing the film thickness and structural integrity. Moreover, the films with nanoparticles exhibited reduced moisture content and solubility, which is beneficial for packaging applications where stability and durability are critical. The films showed a significant reduction in water vapor permeability, indicating that the nanoparticles and spermidine formed an effective barrier against moisture diffusion. Additionally, the XRD and FTIR analyses confirmed the structural changes and interactions within the films, providing further insights into the molecular-level modifications induced by the additives. These results pave the way for developing sustainable and functional packaging materials that meet the increasing demand for eco-friendly solutions in various industries.

## Figures and Tables

**Table 1:** Film thickness, tensile strength (TS), percentage of elongation at break (EAB %), Young’s modulus (Ym).

	Thickness (mm)	Tensile Strength (TS) (MPa)	Elongation (%)	Young’s Modulus (Ym) (MPa)
Control	0.140 ± 0.002 ^c^	9.203 ± 0.257 ^d^	124.123 ± 2.888 ^c^	22.927 ± 0.522 ^c^
CH/G/SPD	0.144 ± 0.004 ^c^	9.327 ± 0.370 ^d^	125.467 ± 2.163 ^c^	24.250 ± 0.171 ^c^
CH/G/SPD/ZnO	0.155 ± 0.003 ^b^	14.073 ± 0.172 ^c^	170.010 ± 1.650 ^b^	52.743 ± 0.444 ^b^
CH/G/SPD/GO	0.154 ± 0.003 ^b^	15.143 ± 0.262 ^b^	174.987 ± 1.840 ^a^	53.877 ± 0.762 ^b^
CH/G/SPD/ZnO/GO	0.171 ± 0.003 ^a^	17.787 ± 0.391 ^a^	167.420 ± 1.906 ^b^	66.617 ± 2.205 ^a^

Mean values and standard deviation are given. Means bearing different superscripts in the same column are significantly different (*p* < 0.05).

**Table 2:** The moisture content (%) and solubility (%) of CH/G films.

	Control	CH/G/SPD	CH/G/SPD/ZnO	CH/G/SPD/GO	CH/G/SPD/ZnO/GO
Moisture	10.54 ± 1.50 ^a^	6.15 ± 0.10 ^b^	2.60 ± 0.71 ^c^	2.80 ± 1.29 ^c^	3.16 ± 0.60 ^c^
Solubility	35.21 ± 0.10 ^b^	65.63 ± 0.05 ^a^	32.12 ± 0.04 ^bc^	32.26 ± 0.10 ^bc^	21.7 ± 0.06 ^c^

Mean values and standard deviation are given. Means bearing different superscripts in the same row are significantly different (*p* < 0.05).

**Table 3:** Color characteristics (L, a*, b*, and ΔE) of films.

	Control	CH/G/SPD	CH/G/SPD/ZnO	CH/G/SPD/GO	CH/G/SPD/ZnO/GO
Light	0.08 ± 0.01 ^b^	0.07 ± 0.01 ^b^	0.03 ± 0.00 ^c^	0.21 ± 0.00 ^a^	0.08 ± 0.01 ^b^
L	85.47 ± 2.75 ^a^	86.17 ± 0.46 ^a^	73.57 ± 4.01 ^b^	72.67 ± 4.35 ^b^	83.53 ± 3.76 ^a^
a*	3.67 ± 1.63 ^a^	3.37 ± 0.46 ^a^	4.70 ± 0.00 ^a^	4.97 ± 0.92 ^a^	4.93 ± 2.51 ^a^
b*	14.10 ± 3.90 ^ab^	12.80 ± 1.18 ^ab^	9.40 ± 0.00 ^b^	9.93 ± 0.92 ^b^	17.23 ± 4.88 ^a^
ΔE	86.83 ± 2.00 ^a^	87.17 ± 0.40 ^a^	74.33 ± 3.95 ^b^	73.53 ± 4.22 ^b^	85.60 ± 2.46 ^a^

Mean values and standard deviation are given. Means bearing different superscripts in the same row are significantly different (*p* < 0.05).

## Data Availability

The original contributions presented in this study are included in the article. Further inquiries can be directed to the corresponding authors.
